# Penternary chalcogenides nanocrystals as catalytic materials for efficient counter electrodes in dye-synthesized solar cells

**DOI:** 10.1038/srep29207

**Published:** 2016-07-06

**Authors:** Faruk Özel, Adem Sarılmaz, Bilal İstanbullu, Abdalaziz Aljabour, Mahmut Kuş, Savaş Sönmezoğlu

**Affiliations:** 1 Department of Metallurgical and Materials Engineering, Faculty of Engineering, Karamanoğlu Mehmetbey University, 70200, Karaman, Turkey; 2 Selcuk University, Advanced Technology Research and Application Center, 42030, Konya, Turkey; 3 Nanotechnology R&D Laboratory, Karamanoğlu Mehmetbey University, 70200, Karaman, Turkey

## Abstract

The penternary chalcogenides Cu_2_CoSn(SeS)_4_ and Cu_2_ZnSn(SeS)_4_ were successfully synthesized by hot-injection method, and employed as a catalytic materials for efficient counter electrodes in dye-synthesized solar cells (DSSCs). The structural, compositional, morphological and optical properties of these pentenary semiconductors were characterized by X-ray diffraction (XRD), Raman spectroscopy, transmission electron microscopy (TEM), energy-dispersive spectrometer (EDS) and ultraviolet-visible (UV–Vis) spectroscopy. The Cu_2_CoSn(SeS)_4_ and Cu_2_ZnSn(SeS)_4_ nanocrystals had a single crystalline, kesterite phase, adequate stoichiometric ratio, 18–25 nm particle sizes which are forming nanospheres, and band gap energy of 1.18 and 1.45 eV, respectively. Furthermore, the electrochemical impedance spectroscopy and cyclic voltammograms indicated that Cu_2_CoSn(SeS)_4_ nanocrystals as counter electrodes exhibited better electrocatalytic activity for the reduction of iodine/iodide electrolyte than that of Cu_2_ZnSn(SeS)_4_ nanocrystals and conventional platinum (Pt). The photovoltaic results demonstrated that DSSC with a Cu_2_CoSn(SeS)_4_ nanocrystals-based counter electrode achieved the best efficiency of 6.47%, which is higher than the same photoanode employing a Cu_2_ZnSn(SeS)_4_ nanocrystals (3.18%) and Pt (5.41%) counter electrodes. These promising results highlight the potential application of penternary chalcogen Cu_2_CoSn(SeS)_4_ nanocrystals in low-cost, high-efficiency, Pt-free DSSCs.

In recent years, dye-sensitized solar cells (DSSCs) have been extensively studied as a alternative to silicon solar cells because they offer high conversion efficiency, low-cost, environmental friendliness and simple fabrication^1^,^2^,^3^,^4^,^5^ Three important components are required to build a typical DSSC: i) a photoanode consisting of an efficient light-harvesting dye with nanocrystalline semiconductor (TiO_2_); ii) an electrolyte with a redox coupled (triiodide/iodide) for effective charge separation; and iii) a counter electrode (CE) with a robust and efficient reduction/oxidation catalyst. Of these components, the counter electrode (CE) is especially critical in the development of DSSCs because it collects the electrons from the external circuit back to the redox electrolyte and catalyzes the reduction of triiodide to iodide ions. Although platinium (Pt) is often considered to be a superior CE material due to its excellent catalytic activity, chemical stability and electrical conductivity, it has some drawbacks such as the limited reserves and high cost which prevents its large scale industrial applications^3^,^4^,^6^,^7^,^8^. Therefore, the development of new materials using cheaper and abundant elements for producing Pt-free CEs for DSSCs as well as the novel design in solar cells such as p-type DSSCs or tandem structures has become technologically desirable.

Thus far, many functional materials have been studied to replace Pt as CEs for cost-effective and high-efficiency DSSCs including carbon-based materials^9^,^10^,^11^ and conjugated polymers^12^,^13^,^14^. However, their stabilities and catalytic activities are not satisfactory, and thus DSSCs fabricated using these materials still suffer from poor thermal stability and weak corrosion resistance. Versus carbon materials and conductive polymers, inorganic materials have unique properties such as material diversity, abundance, low cost, high catalytic activity, and ease of modification. Therefore, extensive research has been performed on using inorganic compounds such as transitional metal carbides, nitrides, oxides, and sulfides as well as ternary/quaternary/penternary chalcogenides as a new class of alternative catalytic materials for Pt in DSSC systems^3^,^4^,^15^,^16^,^17^,^18^,^19^,^20^,^21^.

Currently, chalcogenide nanocrystals (NCs) such as Cu_2_ZnSnS_4_ (CZTS), Cu_2_ZnSnSe_4_ (CZTSe) and new quaternary compositions can replace zinc (Zn^2+^) with other transition metal ions such as Fe^2+^, Ni^2+^ and Mn^2+^ etc. This has attracted research interest as promising candidates for Pt-free counter electrodes because of their excellent catalytic activities, low-cost, good conductivity and environmental stability^3^,^20^,^22^,^23^. Besides quaternary chalcogenide NCs, a fifth metal ion can be incorporated into quaternary chalcogenides and converts penternary chalcogenides such as Cu_2_ZnSn(SeS)_4_ (CZTSeS) and Cu_2_XSn(SeS)_4_ (X = Fe^2+^, Co^2+^, Ni^2+^ and Mn^2+^ etc) which provide additional optical, photochemical, thermoelectric and magnetic functions^24^,^25^,^26^. The first example of a penternary chalcogenides-based counter electrode for DSSCs was developed by Xin and coworkers^4^. This device combines a photoanode consisting of nanoparticulate anatase TiO_2_ sensitized with a ruthenium–based dye and a photocathode consisting of CZTSeS nanoparticle catalyst. Their device has good catalytic performance for regeneration of iodide from triiodide in a redox electrolyte, and has an efficiency of 7.37%, which is even higher than that of the Pt-based DSSCs (7.04%). Although this device is inefficient because it has lower electron transport efficiency, it is a proof-of-concept that DSSCs can be created with penternary chalcogenide-based counter electrodes. While CZTS and CZTSe have been reported, there are few literature reports on the synthesis of both Cu_2_CoSnS_4_ (CCTS) and Cu_2_CoSnSe_4_ (CCTSe) nanocrystals and their properties^24^,^25^,^27^,^28^,^29^. The literature has a surprising lack of details on both the synthesis of penternary Cu_2_CoSn(SeS)_4_ (CCTSeS) NCs and their utility as counter electrodes in DSSCs.

Here, penternary chalcogenides CCTSeS and CZTSeS nanocrystals were successfully synthesized by a hot-injection method and characterized using X-ray diffraction (XRD), Raman spectroscopy, transmission electron microscopy (TEM), energy-dispersive spectrometer (EDS) and ultraviolet-visible (UV–Vis) spectroscopy. Moreover, these penternary chalcogenides were employed as catalytic materials rather than Pt in a conventional DSSC configuration for the first time. The fabricated DSSCs were characterized by *I-V* and electrochemical impedance spectroscopy measurements. The counter electrode-based CCTSeS catalytic material incorporated Co^2+^ ions into the CZTSeS structure substituting for Zn^2+^ ions, enhances the photocurrent in the visible region as well as the efficiency of the cell. The study may facilitate new pathways for highly electrocatalytic DSSC materials.

## Result and Discussions

**Figure 1:**
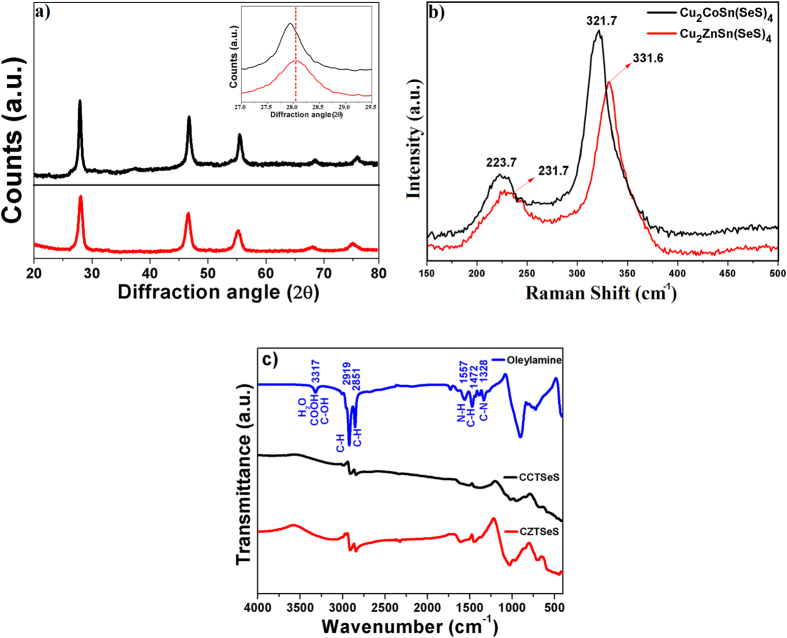
(**a**) XRD Patterns and (**b**) Raman spectrums of Cu_2_CoSn(SeS)_4_ and Cu_2_ZnSn(SeS)_4_ nanocrystals at room temperature. (**c**) FTIR spectra of oleylamine and both nanocrystals.

The XRD patterns of the as-synthesized both nanocrystals are shown in Fig. 1a. The CCTSeS and CZTSeS nanocrystals, having an obvious XRD pattern, show similar patterns. Displacement of the Co atoms with Zn atoms in the lattice structure does not cause any change in the tetragonal arrangement. Three intense peaks at 2ϴ = 28, 45 and 56 correspond to planes (112), (220), and (312), respectively, which are well matched with the standard kesterite pattern (PDF: 26-0575). In this phase, the chalcogen ions (S and Se) and cations (Co^+2^ or Zn^+2^) are arranged in crystallographic c-direction^30^ because sulfur and selenium ions were joined to four cations (in turn joined to four anions). All samples show broad diffraction peaks, and these broad peaks are due to the small size of the both nanocrystals. The average crystallite size of CCTSeS and CZTSeS nanocrystals were calculated from the [112] diffraction peaks by using Scherer’s equation and found to be around 17 ± 5 nm and 19 ± 1 nm, respectively. Furthermore, the (112) peak intensity of CCTSeS nanocrystals are significantly stronger and narrower than CZTSeS nanocrystals. This indicates that CCTSeS nanocrystals have a higher aspect ratio than the CZTSeS nanocrystals. In order to confirm whether Co^+2^ ions are substituted Zn^+2^ ions into the host crystal, we investigated the shift of the XRD peak positions in the inset of Fig. 1a. The diffraction peak of CCTSeS NCs were shifted slightly toward lower 2ϴ side as compared with CZTSeS NCs, indicating the increased lattice constants of CCTSeS NCs due to ionic size differences between Co^2+^ (0.58 Å) and Zn^2+^ (0.60 Å) ions in the same coordination number. The peak position shift of XRD shows that Co^+2^ ions are substituted Zn^+2^ ions into the host crystal.

The structure and phase purity of the both nanocrystals were investigated by Raman analysis. The Raman spectra of these nanocrystals are shown in Fig. 1b. The characteristic peaks of CZTSeS are obvious. Two Raman peaks at 331.6 and 231.7 cm^−1^ corresponded to the A_1_ modes of CZTS and CZTSe, respectively^31^,^32^. The Raman peak at 331.6 is slightly shifted to lower values, while the CZTSe Raman peak at 231.7 shifted to higher frequency direction versus the standard peak positions. This is due to the coexistence of larger Se (1.98 Å) and smaller S (1.84 Å) atoms. Moreover, these peaks indicated that our samples have more sulfur than selenium^32^, which was confirmed by EDS. No Raman peaks were associated with other crystalline forms. This demonstrates that all of the samples high purity and crystallinity. Displacement of the Co and Zn atoms in the lattice structure does not cause any changes in the tetragonal arrangement. However, the CCTSeS shifted to a lower frequency direction versus the CZTSeS peak positions. The values are similar because all of these modes involve only vibrations of the S and Se atoms. Consequently, they are independent from mass effects. Only the dominant peak at higher frequencies shows a certain dependence on the Co–SSe/Zn–SSe force constants^33^,^34^.

In order to confirm whether the organic molecules on the surface of both NCs were completely removed after annealing which is performed to fabricate counter electrodes, Fig. 1c shows the FTIR spectra of CCTSeS and CZTSeS nanocrystals as well as OLA. From Fig. 1c, the characteristic absorption bands of OLA can be clearly identified as follows: at 3317 cm^−1^ corresponding to stretching vibrations of C-OH, COOH, and H_2_O modes, at the range of 2840–3000 cm^−1^ corresponding to C–H symmetric and asymmetric stretching vibrations modes, at range of 1540–1750 cm^−1^ corresponding to N–H bending mode, at 1472 cm^−1^ corresponding to bending vibrations of C–H mode, at 1328 cm^−1^ corresponding to C–N stretching mode and at near 1000 cm^−1^ corresponding to NH_2_ bending modes^21^,^35^. Although the other strong characteristic absorption bands of OLA were not observed on the surface of both NCs, surprisingly, CZTSeS nanocrystals exhibit a weak absorption bands, having highly intense compared to CCTSeS nanocrystal, at near 2851 cm^−1^ and 2919 cm^−1^ corresponding to the vibrations of C–H bonds in OLA adsorbed onto the nanocrystal surfaces. This result showed that CZTSeS nanocrystals have more residual OLA on its surfaces when compared to CCTSeS nanocrystal. As a consequence, not all organic ligands are completely removed on the surface of both NCs after annealing.

**Figure 2:**
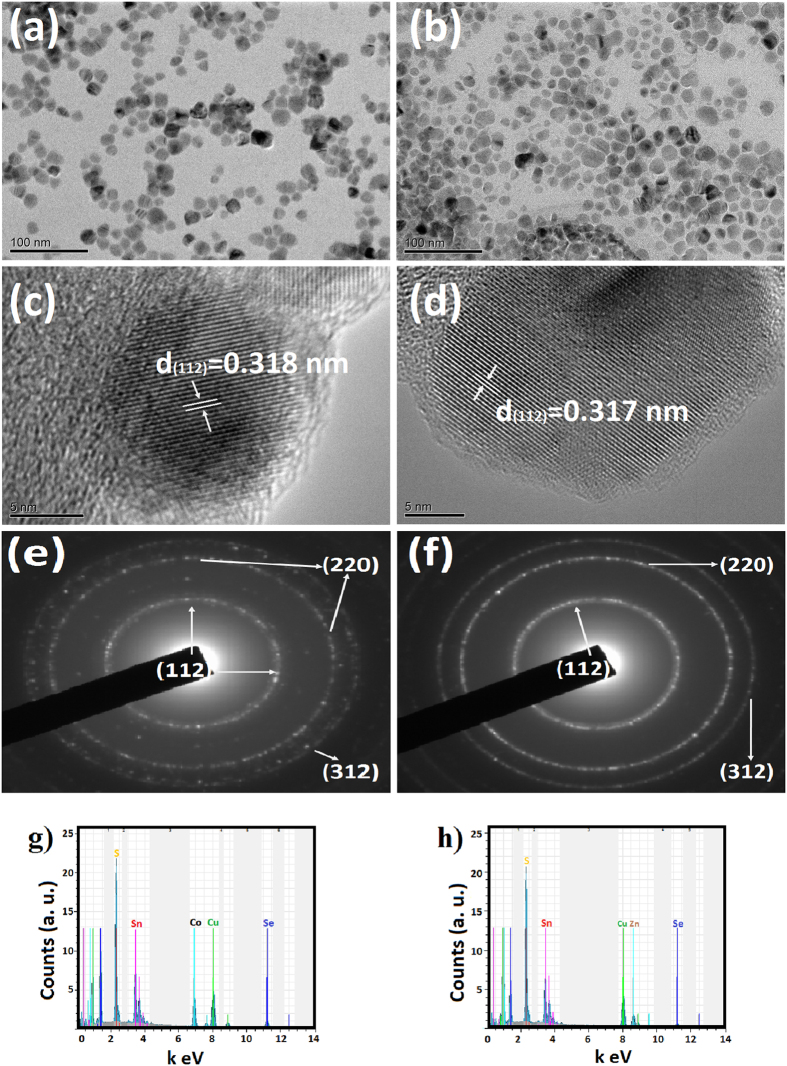
(**a,b**) TEM images, (**c,d**) HR-TEM images, (**e,f**) SAED patterns and (**g,h**) EDS spectra of Cu_2_CoSn(SeS)_4_ and Cu_2_ZnSn(SeS)_4_ nanocrystals, respectively.

Figure 2a–f shows TEM images and selected area diffraction (SAED) patterns of the as-synthesized both nanocrystals. Figure 2(a,b) clearly shows that the nanocrystals are spherical and the CCTSeS and CZTSeS nanocrystals have an average size of 18 and 25 nm, respectively. The standard deviation of these CCTSeS and CZTSeS nanocrystals were ~2 nm and ~5 nm, respectively. In addition, we used HR-TEM to further confirm the crystallinity and structure of the nanocrystals. Figure 2(c,d) gives HR-TEM images of the nanocrystals—the lattice fingers of the nanocrystals revealed their highly crystalline nature. The interplanar spacing of CCTSeS and CZTSeS nanocrystals were measured and found to be 3.18 Å and 3.17 Å, respectively. These were attributed to the (112) crystallographic planes. The SAEDs patterns agree well with the structure of the CCTSeS and CZTSeS as shown in Fig. 2(b,c). The polycrystalline SAEDs are indexed with rings to (112), (220) and (312). These match with the structure of the tetragonal CZTSeS nanocrystals^36^. As shown in Fig. 2(e,f), the diffraction rings are discontinuous and consist of sharp spots, which indicate that the nanocrystals are well crystallized. Although the SAED data suggests that both have good crystallinity, the CCTSeS nanocrystals exhibit better crystalline features than the CZTSeS nanocrystals as confirmed by XRD. The chemical compositions of the these nanocrystals have been estimated from EDS measurements and the EDS spectra is given in Fig. 2(g,h). It can be seen that the average compositions of both nanocrystals were found to be Cu_2_Co_1_Sn_1_(S_0,66_Se_0,34_)_4_, Cu_2_Zn_1_Sn_1_(S_0,74_Se_0,26_)_4_, respectively. These results indicate that both nanocrystals were close to the expected stoichiometry (2:1:1:4) and probably ionic exchanged between Co^2+^ and Zn^2+^ ions was realized.

**Figure 3:**
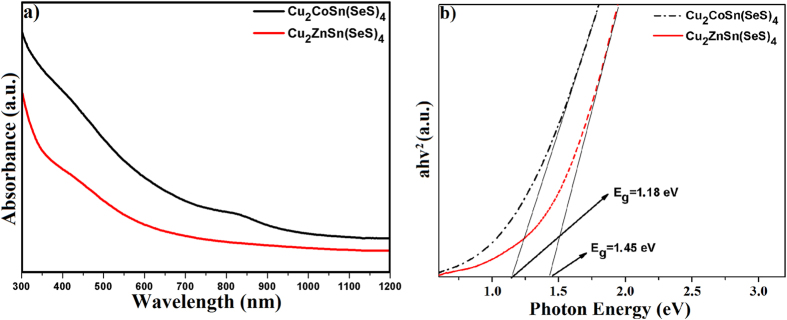
(**a**) UV-Vis absorption spectrums and (**b**) eV Diagrams of Cu_2_CoSn(SeS)_4_ and Cu_2_ZnSn(SeS)_4_ nanocrystals.

The UV-Vis absorption spectra of the as-synthesized nanocrystals are shown in Fig. 3. The CCTSeS and CZTSeS nanocrystals exhibited strong and broad absorption in the UV region with tails extending to the red region. The band gap value was determined by plotting (Ahʋ)^2^ versus hʋ and extrapolating the linear portion of the spectrum in the band edge region. For direct band gap compounds, the band gap can be calculated by plotting the product of photon energy and absorbance squared versus photon energy from the absorbance spectrum and finding the intercept of the abscissa^37^. The optical band gaps of these CCTSeS and CZTSeS nanocrystals were 1.18 and 1.45 eV, respectively. Note that these band gaps are in the range of 1.0–1.5 eV depending on the elemental content of structures. Moreover, the band gap value is close to the optimal values reported in the literature for solar cells^38^.

**Figure 4:**
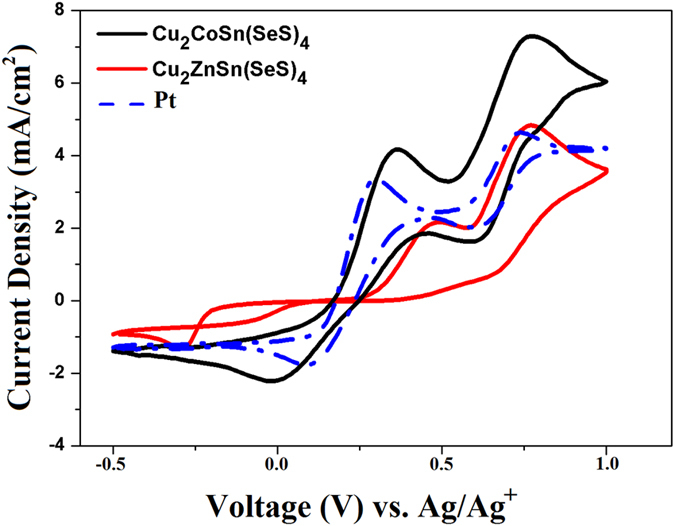
Cylic voltammograms for Cu_2_CoSn(SeS)_4_ and Cu_2_ZnSn(SeS)_4_ nanocrystals and Pt counter electrodes.

Cyclic voltammetry experiments were carried out to investigate the catalytic activities and reaction kinetics of both nanocrystals. Figure 4 shows typical cyclic voltammetry curves of Pt, CCTSeS and CZTSeS nanocrystals performed in a three-electrode system in iodine/iodide electrolyte. Two pairs of redox waves were observed on all of electrodes. These pairs explain the oxidation and reduction of I^−^/I_3_^−^ and I_2_/I_3_^−^, respectively^39^. The reduction peak, known as the cathodic peak, could evaluate the catalytic activity of CEs because the counter electrode in a DSSC serves to catalyze the reduction of I_3_^−^ to I^− ^10,12,39. In Fig. 4, the CCTSeS-based electrode shows a much larger current density for the I_3_ reduction than both the CZTSeS and Pt-based electrodes, which implies a faster redox reaction rate and a better electrocatalytic activity for the I_2_/I_3_^−^ redox couple on the CCTSeS-based CEs. This is due to the increase in crystallinity, the large active surface area and the increased catalytic active sites of the CCTSeS-based counter electrode^40^,^41^,^42^. However, when Zn^+2^ ions are replaced with Co^+2^ ions into the kesterite structure, the CZTSeS electrode shows both lower anodic and cathodic peak current densities than the CCTSeS and Pt–based electrodes. This confirms lower electrocatalytic activity. Although CZTSeS-based electrodes with better catalytic performance than Pt-based electrode have been reported by other researchers^4^, we observed the opposite. This difference was mainly caused by quality of CZTSeS films such as the existence of organic ligands on surface, the worse catalytic properties, its much larger size and lower conductivity of CZTSeS nanocrystal-based electrodes^16^,^21^. They also have weaker adhesion to FTO than that of Pt particles and CCTSeS NCs^26^,^43^. The catalytic activity data implies that CCTSeS nanocrystals could be used as an efficient counter electrode in DSSCs and could replace Pt.

**Figure 5:**
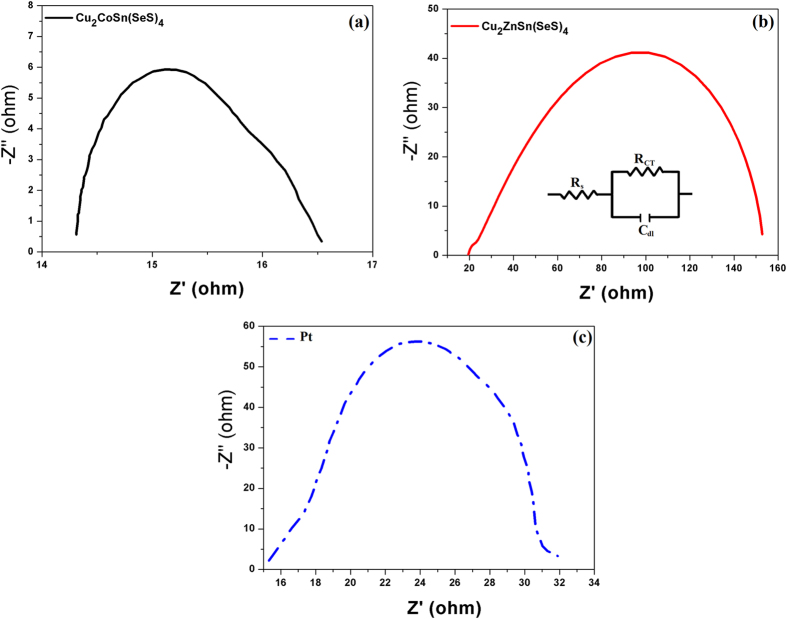
Nyquist plots of as-prepared symmetric cell based on (**a**) Cu_2_CoSn(SeS)_4_, (**b**) Cu_2_ZnSn(SeS)_4_ and (**c**) Pt counter electrodes at open circuit conditions.

To understand the charge transfer process at the interface between the electrolyte and the counter electrode, we measured the EIS with symmetric sandwich-type configuration to eliminate the influence of the photoanode. Electrochemical impedance spectroscopy (EIS) measurements were carried out to compare the charge transfer and ion transport characteristics of the different electrodes. In Fig. 5, the EIS results show well-defined single semicircles over the high frequency range related to the impedance of charge transfer process occurring at the counter electrode and electrolyte interface. The CCTSeS electrode has the lowest charge-transfer resistance (R_CT_) of 2.23 Ω, which is lower than that of Pt (14.75 Ω) and CZTSeS (128.34 Ω) electrodes (see in Table 1). This result suggests higher electrocatalytic activity and lower recombination at the interfaces in DSSCs with CCTSeS NCs as CEs^15^,^44^. In contrast, the CZTSeS electrode exhibited huge R_CT_ values indicating poor catalytic activity that can be attributed to the organic ligand capped on the surface NCs^16^,^21^. Besides the charge transfer impedance, the sheet resistance of the counter electrode (R_s_) is another important parameter affecting the performance of counter electrode and it can also be obtained from the EIS. We found that the CCTSeS electrode has the lowest sheet resistance; the other counter electrodes have similar values. This demonstrates that CCTSeS NCs has a prominent conductivity. However, R_CT_ and R_s_ are not the only deciding factor for counter electrode performance. The double layer capacitance (C_dl_) can also provide information on the catalytic activity of the counter electrode. The C_dl_ of CCTSeS (87 mF) is higher than that of the other CEs and corresponds to the higher surface area, which is a crucial factor for high electrocatalytic activities as confirmed by TEM. In view of the excellent electrocatalytic activity and lower charge transfer resistance, it is expected that a DSSC based on the CCTSeS electrode can achieve improved performance.

**Figure 6:**
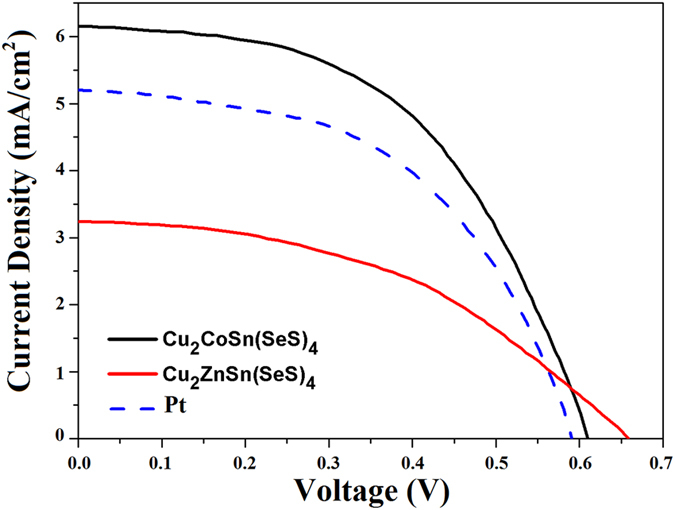
*J–V* characteristics of DSSCs employing Cu_2_CoSn(SeS)_4_ and Cu_2_ZnSn(SeS)_4_ nanocrystals- and Pt-based counter electrodes.

The photocurrent density versus photovoltage (*J–V*) curves of three DSSCs are depicted in Fig.6, and their characteristic parameters, including the short-circuit current (J_sc_), the open-circuit voltage (V_oc_), the fill factor (FF), and the energy conversion efficiency (η), are summarized in Table 1. The DSSC employing CCTSeS as a counter electrode exhibited the best performance with V_oc_ of 0.61 V, J_sc_ of 6.14 mAcm^−2^, FF of 0.57 and an efficiency of 6.47%, which is greater than both 3.18% for CZTSeS and 5.41% for Pt counter electrodes. The significant increase in Jsc and cell efficiency can be explained by the lower charge resistance, removal of organic molecules on surface and enhancement of crystallization^21^,^44^,^45^,^46^. Moreover, it is clear from TEM studies that the particle size of CCTSeS NCs is lower than CZTSeS NCs. The smaller particle sizes provide large surface area and effective path for electrons transportation and redox reaction resulting in smoother and more efficient electron transport in the CCTSeS-based CE. Another reason for the poor performances of CZTSeS-based CEs is the increased charge transfer resistance at the counter electrode and its poor electrocatalytic property that was confirmed by the EIS and CV analysis, respectively. Therefore, CCTSeS-based CE has higher conversion efficiency than CZTSeS-based CE. In short, the CCTSeS counter electrodes, having superior electrocatalytic activity to reduce oxidized triiodide to iodide, exhibit the best performance as an efficient alternative material between others.

## Conclusions

In summary, we synthesized penternary chalcogenides CCTSeS and CZTSeS nanocrystals with a hot-injection method, and investigated their application as counter electrodes in DSSCs. The XRD and TEM results revealed that both CCTSeS and CZTSeS nanocrystals are kesterite and are approximately 18–25 nm particles. The CV and EIS results show that the CCTSeS nanocrystals have better electrocatalytic properties than both CZTSeS nanocrystals and Pt. Furthermore, the CCTSeS nanocrystals can be counter electrodes in the fabrication of DSSCs, resulting a power conversion efficiency of 6.47% versus Pt-based and CZTSeS-based CEs of 5.41% and 3.18%, respectively. This efficiency was the first report using CCTSeS nanocrystals as CEs. Consequently, the excellent electrocatalytic properties, facile synthesis and low-cost of the CCTSeS nanocrystals may make large-scale production of DSSCs feasible.

## Experimental Section

### Synthesis of CMTSeS Nanocrystals

The synthesis of CCTSeS and CZTSeS nanocrystals used a previously published hot-injection process with metal salts and oleylamine (OLA) precursors as starting materials and capping agent, respectively^47^,^48^. Typically, 2 mmol cupper(II) acetate monohydrate, 1 mmol M (II) acetate (Zinc(II) acetate dihydrate or cobalt(II) acetate tetrahydrate) were added separately. Depending on the desired chemical structure, 1 mmol tin(II) acetate and 20 mL OLA were added to a 25 mL two-neck flask and heated to 280 °C under N_2_. The colors changed from blue to reddish brown and a solution of sulfur in oleylamine was added. The reaction was continued for 30 min at 280 °C. After 30 min with continuous stirring, the reaction mixture was cooled to room temperature. Finally, both nanocrystals were precipitated by adding toluene and 2-propanol (4:1) mixtures and then centrifuged. The final product was washed with ethanol and dried at 70 °C.

### Fabrication of Dye-Sensitized Solar Cells

We manufactured TiO_2_ nanoparticle-based paste according to ref. 49. In a typical procedure, the TiO_2_ paste were made from 21-nm TiO_2_ nanopowder in ethanol and printed to FTO substrates using the doctor blade technique. All films were annealed at 450 °C for 1 h before sensitization. The thickness and active area of the TiO_2_ films were approximately 10 μm and 1 cm^2^, respectively. To fabricate the working electrodes (photoanodes), the TiO_2_ films were immersed in a dye solution (3.0 × 10^−4^ M mixture of the ruthenizer 535-bisTBA (N-719) in methanol) for 12 h. The iodine/iodide (I^−^/I_3_^−^) electrolytes and Pt counter electrodes were prepared according to our previous report^50^. To prepare counter electrodes (photocathodes), the as-synthesized both nanocrystals were dissolved in toluene at the same concentration and then stirred with a magnetic stirrer for 2 h at room temperature. Before deposition, the FTO glasses (Asahi Glass, fluorine-doped SnO_2_; sheet resistance: 15 sq^−1^) were cleaned in a detergent solution in an ultrasonic bath for 15 min, rinsed with water and ethanol and then dried. Next, a spin coating process was used to grow the prepared solutions on FTO glass substrates at 2000 rpm for 30 s. This process was repeated 15 times. Finally, the resulting CCTSeS and CZTSeS-based counter electrodes were annealed at 150 °C for 10 min in a furnace.

We fabricated the device according to the following method. The photoanode was placed face up on a flat surface, and the catalyst-coated counter electrode was placed on top of the photoanode. These two opposing glass plates were offset from one another so that the entire photoanode was covered by the counter electrode. The I^−^/I_3_^−^ electrolyte solution was placed at the edges of the plates, and the liquid was drawn into the space between the electrodes via capillary action. We used an epoxy adhesive to hold the electrodes together. Accordingly, we fabricated the CCTSeS, CZTSeS and Pt (as a reference device) counter electrode-based DSSCs.

### Instrumentation

The X-ray diffraction (XRD) pattern of the nanocrystals was recorded with a Bruker Advance D8 diffractometer (Cu a source with 1.5406 wavelength) in powder mode. The morphology and composition of these nanocrystals was analyzed by JEOL JEM-2100F 200 kV model transmission electron microscopy (TEM) and ZeissEvo model energy-dispersive spectroscopy (EDS), respectively. The ultraviolet (UV)-visible absorption measurements were conducted using Biochrom Libra S22 spectrometer. Fourier transform infrared (FTIR) spectra were recorded on a Bruker-Vertex 70 spectrometer in the range 4000-400 cm^−1^. Cyclic voltammetry (C–V) studies obtained at a scan rate of 50 mV/s in electrolyte solution composed of 10 mM LiI, 1 mM I_2_ and also 0.1 M LiClO_4_ as supporting electrolyte in acetonitrile and electrochemical impedance spectroscopy (EIS) were carried out by an Ivium-compactStat model potentiostat/galvanostat in a three-electrode configuration. The current-voltage (I-V) curves of DSSCs were recorded using a Keithley 4200 SCS characterization system and standard solar irradiation of 30 mWcm^–2^ (xenon arc lamp with AM 1.5 filter) as the light source (Solar Light XPS 300 solar spectrum).

## Additional Information

**How to cite this article**: Özel, F. *et al*. Penternary chalcogenides nanocrystals as catalytic materials for efficient counter electrodes in dye-synthesized solar cells. *Sci. Rep.* **6**, 29207; doi: 10.1038/srep29207 (2016).

## Figures and Tables

**Table 1:** The photovoltaic and EIS parameters of the DSSCs based on Cu_2_CoSn(SeS)_4_ and Cu_2_ZnSn(SeS)_4_ nanocrystals and Pt counter electrodes.

Counter electrodes	J_sc_ (mAcm^−2^)	V_oc_ (V)	FF	η (%)	R_s_ (Ω)	R_CT_ (Ω)	C_dl_ (mF)
Cu_2_ZnSn(SeS)_4_	3.23	0.65	0.45	3.18	19.30	128.34	0.0006
Cu_2_CoSn(SeS)_4_	6.14	0.61	0.57	6.47	14.31	2.23	87
Pt	5.19	0.59	0.53	5.41	15.20	14.75	1.22
